# Evaluation of GLP-1 receptor agonist therapy in the management of steroid-induced diabetes: a narrative review

**DOI:** 10.3389/fcdhc.2026.1772391

**Published:** 2026-04-10

**Authors:** Aleksandra Jędrasek, Karolina Lisowska, Leszek Czupryniak

**Affiliations:** 1 Department of Diabetology and Internal Medicine, Medical University of Warsaw, Warsaw, Poland; 2 Doctoral School, Medical University of Warsaw, Warsaw, Poland

**Keywords:** GIP/GLP-1 receptor agonist, glucagon-like peptide 1 receptor agonist, glucocorticosteroids, semaglutide, steroid-induced diabetes, steroid-induced hyperglycaemia

## Abstract

Glucocorticosteroids (GCs) are commonly used in the treatment of autoimmune, inflammatory and neoplastic diseases. Although clinically effective, they are associated with significant metabolic side effects, including increased insulin resistance, impaired function of pancreatic *β*-cells, and, finally, weight gain. These effects can result in steroid-induced hyperglycemia (SIH) and steroid-induced diabetes (SID), both of which increase the risk of complications such as infections and prolonged hospitalisation. Intensive insulin therapy remains the standard treatment for SID. However, emerging evidence suggests that glucagon-like peptide 1 receptor agonists (GLP-1 RAs) may have therapeutic potential to counteract the metabolic effects of steroids. This review evaluates the efficacy and safety of GLP-1 RAs for the treatment of SIH and SID.

## 1 Introduction

Glucocorticosteroids (GCs) are commonly used to treat inflammatory, neoplastic, and allergic diseases and are part of immunosuppression therapy after transplantation. Despite their therapeutic efficacy, they are associated with metabolic side effects, most significantly steroid-induced hyperglycaemia (SIH) and steroid-induced diabetes (SID). SIH refers to elevated blood glucose levels during GCs therapy and includes both patients without and with pre-existing type 2 diabetes mellitus (T2DM). SID is diagnosed in individuals without a prior history of T2DM, who meet standard diagnostic criteria for diabetes during glucocorticoid exposure (1, 2).

**Figure 1:**
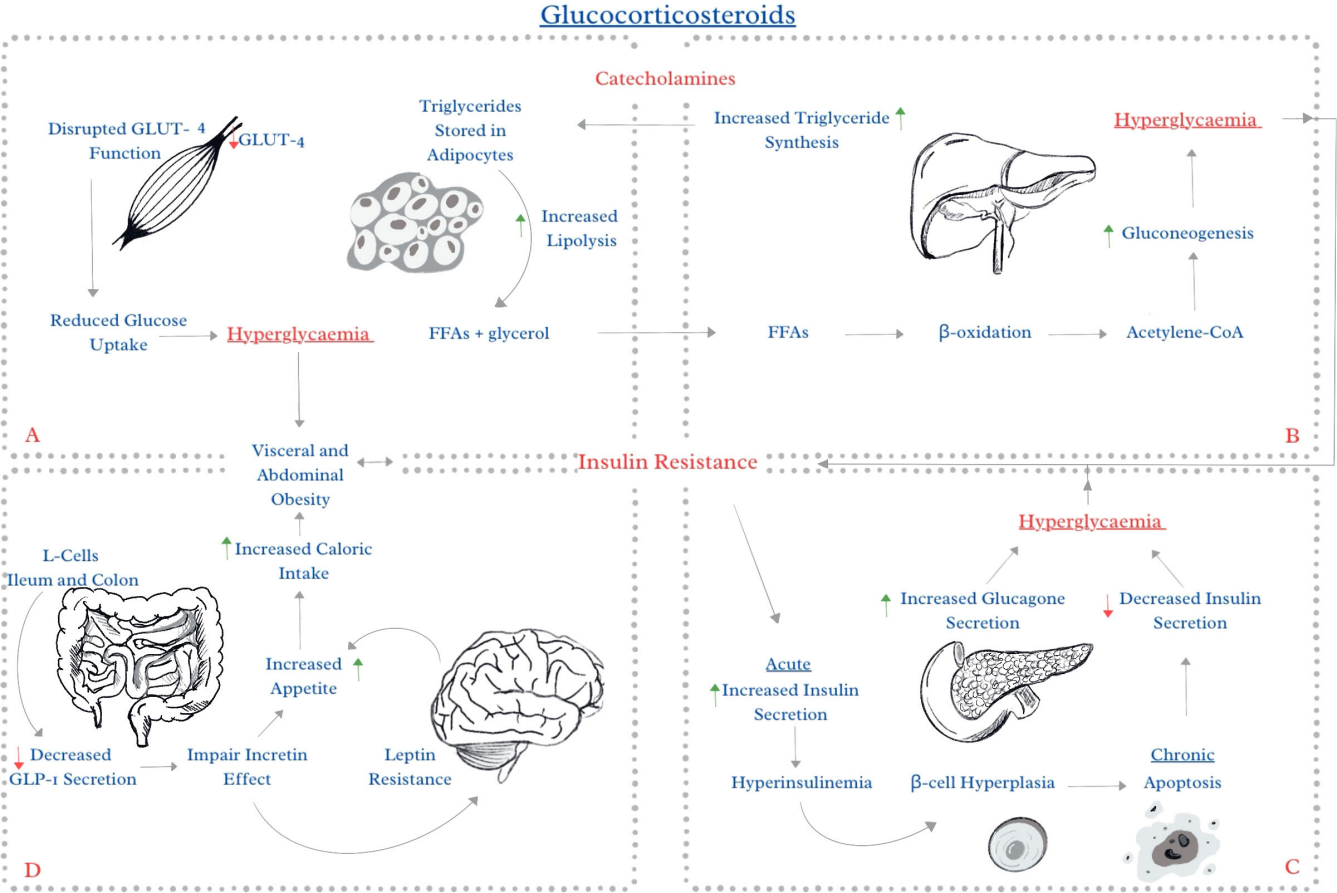
Metabolic effects of glucocorticoids. GCs increase the diabetogenic effect of catecholamines. A central feature of steroid-induced hyperglycaemia is insulin resistance in the liver, skeletal muscle, and adipose tissue, driven primarily by increased hepatic glucose production, lipolysis, and elevated circulating free fatty acids. **(A)** GCs reduce peripheral glucose uptake by disrupting GLUT4 function in muscle and adipose tissue. They increase lipolysis, leading to an increase in FFAs. **(B)** Excess FFAs entering the liver stimulate *β*-oxidation and increase acetyl-CoA levels, which activate gluconeogenesis. Due to increased insulin resistance, the suppression of gluconeogenesis by insulin is ineffective. **(C)** At the pancreatic level, GCs directly impair *β*cell function, promote endoplasmic reticulum stress, lipid accumulation, and apoptosis, leading to reduced insulin secretion. Simultaneously, GCs increase *α*-cell glucagon secretion, collectively exacerbating hyperglycaemia. **(D)** The glucocorticoid receptor is present in GLP-1-producing cells, and its activation diminishes the secretory responsiveness of these cells. GCs impair incretin effect, increase appetite, leading to excessive caloric intake. GCs stimulate adipogenesis and fat redistribution, favouring visceral and abdominal adiposity. This vicious circle leads to significant hyperglycaemia and further increased insulin resistance. FFAs, free fatty acids; The figure was created by the author (AJ).

The strongly diabetogenic effect of GCs is caused by increased insulin resistance, driven primarily by increased hepatic gluconeogenesis, lipolysis, and elevated circulating free fatty acids (3–5). GCs reduce peripheral glucose uptake, promote apoptosis in *β*-cells and increase *α*-cell glucagon production, collectively exacerbating hyperglycaemia (6–9). Furthermore, GCs stimulate adipogenesis and fat redistribution, favouring visceral and abdominal adiposity (9, 10). Recent studies have examined the effects of GCs on incretin hormone regulation and appetite stimulation as a potential novel therapeutic target (11, 12). A simplified summary of the metabolic effects of GCs is presented in **Figure 1**. These mechanisms occur within hours after GCs administration and may lead to hyperglycemia, even in individuals without a prior history of glucose abnormalities (13). Even low doses of GCs are proven to impair glucose metabolism and cause additional metabolic complications (14).

The clinical impact of hyperglycemia during GCs therapy has been well established, with multiple studies reporting increased morbidity and mortality, including higher rates of infection, cardiovascular events and prolonged hospitalisations (8, 15). Patients may develop SIH within hours after GCs administration. In addition, the SID is characterised by a rapid and dynamic increase in body weight. The optimal medical management of SIH and SID is not definitively specified, and insulin therapy remains the standard of care (16). However, its use requires daily injections, is associated with a significant risk of hypoglycaemia, and exerts anabolic effects that, particularly when combined with GCs therapy, may lead to progressive weight gain.

Although glucagon-like peptide-1 receptor agonists (GLP-1 RAs) are not currently recommended in SID by major guidelines, their mechanism of action makes them promising candidates to counteract the metabolic impacts of steroids. Compared with insulin therapy, they achieve similar overall glycemic control, while avoiding insulin-associated adverse effects in T2DM (17). Moreover, they provide effective body-weight control and may prevent the development of steroid-induced obesity (17).

## 2 Methodology

This review aims to evaluate the efficacy and safety of GLP-1 RAs in the treatment of SID. A narrative review was conducted based on a literature search for randomised controlled trials, cohort studies, case reports, and systematic reviews reporting outcomes of GLP-1 RAs therapies in SIH and SID.

## 3 Epidemiology

Nearly 1% of the adult population is treated with GCs, with the frequency increasing up to 2.5% among patients between 70 and 79 years of age (3, 18). The diagnosis of SID is made based on standard diabetes thresholds (19). However, it is important to note that the glycemic profile of steroids is characterised by postprandial hyperglycemia, which may be missed if only fasting glucose or HbA1c is monitored. The recommendations for glucose monitoring during GCs therapy are limited and non-specific (19). In many cases, patients are not adequately evaluated before initiating GCs therapy or during its course (20, 21). Hyperglycaemia during systemic GCs therapy occurs in the majority of hospitalised patients; however, the reported incidence varies widely from 1.5 up to 47% depending on the monitoring protocol (8, 10). A study by Liu et al. reported 32.3% cases of SIH and 18.6% cases of SID among patients without pre-existing diabetes (22). Another meta-analysis of 118 studies established the prevalence of hyperglycaemia during steroid exposure at 10% (23). However, these numbers increase in the settings of continuous glucose monitoring (CGM). Among dermatological patients monitored with CGM sensors from the beginning of GCs therapy, as many as 47.1% were diagnosed with SIH (24).

SIH occurs most frequently in patients receiving steroid therapy for respiratory diseases, with a reported prevalence of 22% (23). However, GCs are administered primarily during acute exacerbations of respiratory disorders, and in many cases, the associated metabolic disturbances are transient. Therefore, a systematic evaluation of patients with SID is important, especially during periods when steroid doses are modified. The risk of persistent glycemic disorders and SID increases with long-term or high-dose GCs use, mainly in oncological patients and patients treated for rheumatic and connective tissue disorders (25).

## 4 Role of GLP-1 receptor agonist therapy in SID

Recently, GLP-1RAs have become an important class of drugs in the management of T2DM, as they significantly lower fasting plasma glucose. Since the insulinotropic activity of GLP-1 RAs is glucose- dependent, they do not pose a significant risk of hypoglycaemia. Subsequently, their potential beyond glycaemic control became the subject of many studies. GLP-1RAs have been proven to reduce the risk of major adverse cardiovascular events (MACE) occurrence, including death from cardiovascular causes, non-fatal myocardial infarction and non-fatal stroke, as well as all-cause mortality in T2DM and patients with obesity (26–28). They also improve kidney outcome by decreasing the development of microalbuminuria and slowing the progression to end-stage kidney disease in T2DM (29). Additionally, GLP-1RAs were recognised for their remarkably significant impact on weight reduction in both diabetic and non-diabetic overweight and obese patients (30). GLP-1RAs are still under investigation for other pathologies. Promising results have been achieved in their implementation in non-alcoholic fatty liver disease and neurodegenerative disorders, as well as in alcohol dependency therapy (31, 32).

The glucocorticoid receptors are present in GLP-1-producing cells, and their activation diminishes the secretory responsiveness of these cells (33). In the animal model, GCs administration decreased the mRNA expression of preproglucagon, the precursor of GLP-1 (10, 11). In a human study, where healthy individuals were exposed to dexamethasone, the authors reported no significant changes in incretin hormone levels after single steroid administration; however, the incretin effect was reduced with increased insulin resistance, even while normal glucose tolerance was maintained (12).

### 4.1 Efficacy of GLP-1 receptor agonist therapy in SID

Considering their beneficial effects on body weight, effective glycemic control, and low risk of hypoglycaemia, GLP-1 RAs may be suitable therapeutic options for SID and SIH, either as adjunctive therapy or as primary treatment. However, they are currently not recommended by major guidelines in these groups of patients. Several studies have examined the potential effects of GLP-1 RAs in SIH and SID; to date, no studies have investigated the role of dual glucose-dependent insulinotropic polypeptide (GIP)/GLP-1 RAs in SIH or SID.

The following sections examine the available evidence addressing glucose control, weight control, safety and all-cause mortality in SID and SIH patients treated with GLP-1 RA.

#### 4.1.1 Glycemic control

In a pioneering study by van Raalte et al. GLP-1 administration successfully prevented glucocorticoid-induced glucose intolerance in healthy men after prednisolone administration (34). The study also demonstrated the protective effect of exenatide on islet-cell function during GCs therapy, providing early evidence for using GLP-1 RAs to counteract the diabetogenic effects of GCs (34). The primary outcome of most studies evaluating GLP-1 RAs therapy in SID and SIH is glycaemic control. Available evidence consistently demonstrates that GLP-1 RAs effectively improve blood glucose regulation in patients receiving glucocorticoid therapy, with significant reductions in HbA1c observed following treatment (35–37). In a study by Pu et al., oncological patients with T2DM, treated with GCs and liraglutide, reported significantly better postprandial glucose levels compared with those receiving insulin therapy alone (38). When used in combination with insulin in hospitalised patients with SIH, GLP-1 RAs were associated with a reduction in total daily insulin dose and injection frequency (39). Similarly, a case series of four patients treated with exenatide reported improved glucose control and HbA1c after four months of therapy (40). In individual cases where insulin therapy was discontinued due to insulin allergy (41) or cognitive decline (42), treatment with GLP-1 RAs (liraglutide and dulaglutide, respectively) successfully achieved satisfactory glycaemic control (41, 42). In head-to-head comparisons, patients with SIH on prednisolone who received dulaglutide for twelve weeks experienced a significantly greater reduction in HbA1c (-0.78; p = 0.034) and total daily insulin dose (-0.29; p = 0.001) compared with sitagliptin (43).

#### 4.1.2 Weight control

The steroid-associated weight gain is a common adverse effect of chronic steroid therapy, leading to further complications. Body weight control emerged as one of the most important outcomes of GLP-1 RAs therapy in SID, a benefit that is rarely achieved with other antihyperglycaemic treatments. This is particularly relevant when compared with intensive insulin therapy, which is frequently associated with weight gain. In the previously mentioned case series, insulin therapy effectively improved glycaemic control; however, it was accompanied by significant weight gain (40). This effect was overcome by shifting to exenatide (40). Similarly, in a cohort study including oncological patients with T2DM and SIH, the group treated with insulin glargine reported significantly higher body mass index (BMI) values compared with those treated with liraglutide (38). This metabolic advantage is notable in the context of excessive complications associated with obesity.

#### 4.1.3 Safety of GLP-1 receptor agonist therapy in SID

GLP-1 RAs and dual GIP/GLP-1 RAs are proven safe in T2DM (29). The most recognised adverse effects are gastrointestinal disturbances, such as nausea, vomiting, constipation, gastritis, and diarrhoea. The severity of these symptoms is mainly mild to moderate, and they are most prominent at treatment initiation and intensification (44). To reduce the risk of gastrointestinal side effects, a gradual escalation of doses is recommended. Among patients treated with semaglutide, about 15-20% experience chronic gastrointestinal adverse effects (45, 46). These adverse effects remain the most common reason for discontinuation of GLP-1 RAs therapy (46). In studies including patients with SID, the prevalence of gastrointestinal side effects did not differ from the T2DM population (39, 47). Especially noteworthy is a study by Pu et al., 131 which enrolled patients with malignant tumours treated with GCs. In this vulnerable population, only 8.47% participants receiving liraglutide reported gastrointestinal side effects (38). Furthermore, the authors did not report any other adverse side effects, and in comparison to the control group treated with insulin therapy, the risk of hypoglycemia was significantly lower (p=0.019) (38). Studies including post-transplant diabetes mellitus (PTDM) patients reported similar results (36, 37). Overall, the GLP-1 RAs therapy in patients with SIH and SID was not associated with increased episodes of hypoglycaemia; moreover, when added to insulin therapy, the risk of these complications was significantly lower, most probably due to decreased insulin doses (39, 43).

The most significant concern regarding GLP-1 RAs therapy in patients receiving GCs is the potential for impaired absorption of medications due to slowed digestion and gastric emptying. This is especially important in the context of transplant recipients and oncological patients. Furthermore, an increase in the intragastric pH, observed during GLP-1 RAs therapy, may also alter the absorption of medications (48). However, diabetes and obesity themselves are associated with altered gastric digestion, which may affect drug absorption (49). Currently number of studies investigated tacrolimus levels during GLP-1 RAs therapy (36). Most data suggested that GLP-1 RAs do not affect tacrolimus levels or transplant outcomes in solid organ transplant recipients (36, 50). Additionally, in many studies, GLP-1 RAs therapy among transplant recipients was well tolerated and demonstrated a safety profile regarding graft function (50–52). However, more long-term, controlled studies are needed to establish the safety of the proposed therapy.

#### 4.1.4 Mortality and adverse events

The available evidence demonstrates that GLP-1 RAs therapy reduces all-cause mortality in T2DM, including patients with chronic kidney disease and increased cardiovascular risk (53). However, only a limited number of studies have evaluated clinical outcomes specifically in patients with SID and SIH, as distinct subgroups. The most relevant study addressing this issue is a large retrospective cohort study by Lin et al. involving adult kidney transplant recipients. Among patients diagnosed with diabetes either before or after kidney transplantation, 78.4% in the GLP-1 RAs group and 79.8% in the control group were receiving steroid therapy (54). The authors reported a significantly decreased risk of all-cause mortality (aHR=0.39; 95% CI 0.31-0.50; p<0.001), lower risk of cardiac arrest and cardiogenic shock (aHR=0.30; 95% CI 0.15-0.58; p=0.001) in the group treated with GLP-1 RAs (54). Concerning renal outcomes, several studies have reported improvements in estimated glomerular filtration rate (eGFR) and reductions in proteinuria among patients treated with GLP-1 RAs (35, 54). In a meta-analysis of cohort studies that reported on outcomes of GLP-1 RAs in adult kidney transplant recipients, no significant changes in eGFR (95% CI 0.64-0.50; p=0.814) or serum creatinine levels (95% CI 0.44-0.28; p=0.668) were observed (36). Only the urine protein to creatinine ratio showed a significant improvement (95% CI 0.77-0.18; p=0.002) (36). In another meta-analysis by Aliyeva et al. the authors reported a significant reduction in all-cause mortality (p=0.009) in the group of transplant recipients receiving steroids, treated with GLP-1 RAs, indicating a 38% lower risk of death compared to nonusers (55). Furthermore, the risk of major adverse kidney events was also significantly lower for GLP-1 RAs users. (p<0.00001) (55). The main limitations of these studies were the high heterogeneity among the included studies and the incomplete reporting of steroid exposure, including dosage and duration. Consequently, the applicability of these findings to patients with SID and SIH remains limited.

Patients receiving GC therapy represent a highly heterogeneous group, including individuals with SID, short-term hyperglycemia in previously normoglycemic patients initiating GC therapy, SIH in patients with T2DM and PTDM. The majority of evidence regarding GLP-1 RAs therapy in patients with steroid-induced dysglycemia originates from cohort studies conducted in solid organ transplant recipients and oncological populations, with a limited number of case reports involving patients with rheumatic diseases. However, in the various populations with steroid-induced dysglycemia, GLP-1 RAs have demonstrated efficacy in improving glycemic control and supporting weight reduction, with additional reported renal and cardiovascular benefits. Importantly, the prevalence of adverse effects does not appear to differ from that observed in patients with T2DM treated with GLP-1 RAs. The summary of studies regarding patients with SID and SIH treated with GLP-1 RAs is presented in **Table 1**.

**Table 1:** Summary of studies reporting efficacy and safety of GLP-1 RAs therapy in SIH and SID.

Study	Design	Population	GC Therapy	Intervention	Main results
Suyama et al. (58) (2024, Japan)	Case series	8 patients with non-Hodgkin lymphoma; SIH	Prednisolone 100 mg × 5 days/cycle	Dulaglutide 0.75 mg/week	↓HbA1C decreased after GLP-1 RA initiation -median 5.9%; ↓ decrease in body weight (p=0.0006); ↓ decreased glycoalbumin levels. (*source not peer-reviewed)
Pu et al. (38) (2022, China)	Cohort study	T2DM patients with malignant tumours (n=60 GLP-1 vs 60 insulin); SIH	N/A	Liraglutide 0.6–1.8 µg/d	↓Lower hospitalisation rate and shorter duration, Lower hypoglycemia rate (p<0.05) in liraglutide group; improved HbA1c, BMI and *β* cell function after 6 months of liraglutide treatment.
Zhang et al. (41) (2021, China)	Case report	SID; relapsing- remitting multiple sclerosis	Methylprednisolone 500 mg i.v. → 6 mg p.o.	Liraglutide 0.6 mg/d + insulin + metformin	Insulin discontinued after 2 months; HbA1c dropped substantially from 12.4% to 8.6%; decrease in body weight by 6.8 kg after 4 months; no significant gastrointestinal symptoms.
Uchinuma et al. (39) (2020, Japan)	Retrospective cohort	Hospitalised patients with SIH (n=38 GLP-1 vs n=38 insulin)	GCs including pulse therapy	Dulaglutide 0.75 mg/week + insulin	↓ Decreased injection frequency (p<0.001) and total daily insulin dose (p<0.01) in the GLP-1 group; no increase in hypoglycemia or GI adverse events.
Hamasaki et al. (42) (2018, Japan)	Case report	SIH; Chronic hypersensitivity pneumonitis	Prednisolone 25mg/d p.o.→ 15mg/d p.o.	Dulaglutide + mitiglinide + insulin	Discontinuation of insulin therapy; improved fasting and postprandial glucose, improvement in C-peptide and glucagon levels.
Matsuo et al. (40) (2013, Japan)	Case series	T2DM patients experiencing SIH; rheumatoid arthritis (2); myasthenia gravis (1); amyopathic dermatomyositis (1)	2: Prednisolone 7mg/d p.o. 1: Prednisolone 10mg/d 1: Prednisolone 2mg 2 ×/d	Exenatide 5 µg twice/d *one patient switched from liraglutide	Improved glycemic control, systolic blood pressure; decrease in low-density lipoprotein cholesterol and triglyceride levels; decrease in body weight in all cases.

SIH, steroid-induced hyperglycaemia; GLP-1 RA, glucagon-like peptide-1 receptor agonist; T2DM, type 2 diabetes mellitus; N/A, not available; /d, per day; BMI, body mass index; SID, steroid-induced diabetes; i.v., intravenous; p.o., per os (oral administration).

## 5 Discussion

SID is a frequent complication of GCs therapy, with limited guidance regarding optimal management strategies. There is a lack of clear recommendations and evidence concerning the optimal timing for initiation of antihyperglycaemic therapy. Furthermore, even after the initiation of insulin therapy, uncertainty remains regarding which additional antihyperglycaemic agents should be introduced and at what stage, especially in the context of long-term GCs treatment. In the era of personalised medicine, it is therefore essential to consider the full range of available therapeutic options and to individualise treatment according to the patient’s clinical profile. The potential to prevent the additional metabolic complications induced by GCs therapy, including *β*-cell dysfunction, insulin resistance, and weight gain is especially important. GLP-1RAs represent one of the most significant pharmacological innovations in recent years. The extensiveness of their overall health benefits has made them one of the primary therapeutic choices for T2DM patients. Beyond glycaemic control, GLP-1RAs have transformed the pharmacological management of obesity and reshaped current understanding of its pathophysiology. More recently, dual GIP/GLP-1 RAs, such as tirzepatide, as well as emerging dual and triple incretin-based therapies, have demonstrated substantial efficacy in both glycaemic and weight control. However, evidence specifically addressing their use in SIH and SID remains limited, underscoring the need for focussed investigation in this patient population.

A notable limitation of these agents is the need for continuous treatment, as discontinuation has been associated with rebound weight gain (56). In patients with T2DM, adherence to GLP-1 RAs therapy is often suboptimal, although it tends to be higher among those receiving once-weekly formulations (57). Additional restrictions to the widespread use of GLP-1 RAs and dual GIP/GLP-1 RAs include high treatment costs and limited accessibility in many healthcare systems. In the hospital settings, the implementation of GLP-1RAs may be particularly challenging, especially with once-weekly formulations, due to the potential need for discontinuation before surgical or diagnostic procedures or in the event of clinical deterioration. In patients receiving variable doses of GCs, intensive insulin therapy offers greater flexibility, as insulin dosing can be rapidly adjusted in response to changing steroid requirements.

The addition of GLP-1 RAs to insulin therapy may allow for improved glycaemic control and, in selected cases, potential de-escalation or discontinuation of insulin. Furthermore, fixed-ratio combination therapies comprising long-acting insulin and GLP-1 RA can integrate the metabolic advantages of GLP-1 RA within the established framework of insulin therapy, while allowing simplified treatment regimens administered as once-daily injections. As our understanding of glucocorticoid-related metabolic dysregulation evolves, therapies targeting the underlying pathophysiological mechanisms offer significant advantages beyond glucose lowering alone. Although insulin remains the standard treatment, GLP-1 RAs may be beneficial for patients with SIH and SID, especially with regard to the potential prevention of glucocorticoid-associated weight gain.

### 5.1 Limitations

Most of the studies yet performed regarding SID and SIH employed observational designs, and data derived from randomised controlled clinical studies are lacking. The existing randomised controlled trials predominantly include healthy participants exposed to glucocorticoids for short durations, which limits their external validity and applicability to routine clinical practice. The marked heterogeneity of patients receiving GCs therapy further complicates the evaluation of the effects of GLP-1 RAs in this population. The majority of available studies have focussed on cohorts of patients with new-onset diabetes following solid organ transplantation or on oncological patients. These groups frequently receive additional medications that independently contribute to hyperglycaemia, thereby confounding the observed effects. Consequently, the implementation of these findings to the broader population of patients who develop metabolic complications secondary to glucocorticoid therapy remains limited.

## 6 Conclusions

GLP-1 RAs may represent a potential therapeutic option for SIH and SID. However, the current evidence base is limited, consisting largely of observational studies in heterogeneous populations. Further randomised controlled trials in representative steroid-treated populations are required to establish optimal treatment timing, monitoring and long-term safety.
