# Supplementary material for: Evaluation of GLP-1 receptor agonist therapy in the management of steroid-induced diabetes: a narrative review
Source: Front Clin Diabetes Healthc. 2026 Apr 10;7:1772391. doi: 10.3389/fcdhc.2026.1772391 (PMC13105962; doi:10.3389/fcdhc.2026.1772391)
Supplement: Supplementary file 1 [file DataSheet1.pdf]

Supplementary Material

1 SUPPLEMENTARY FIGURE

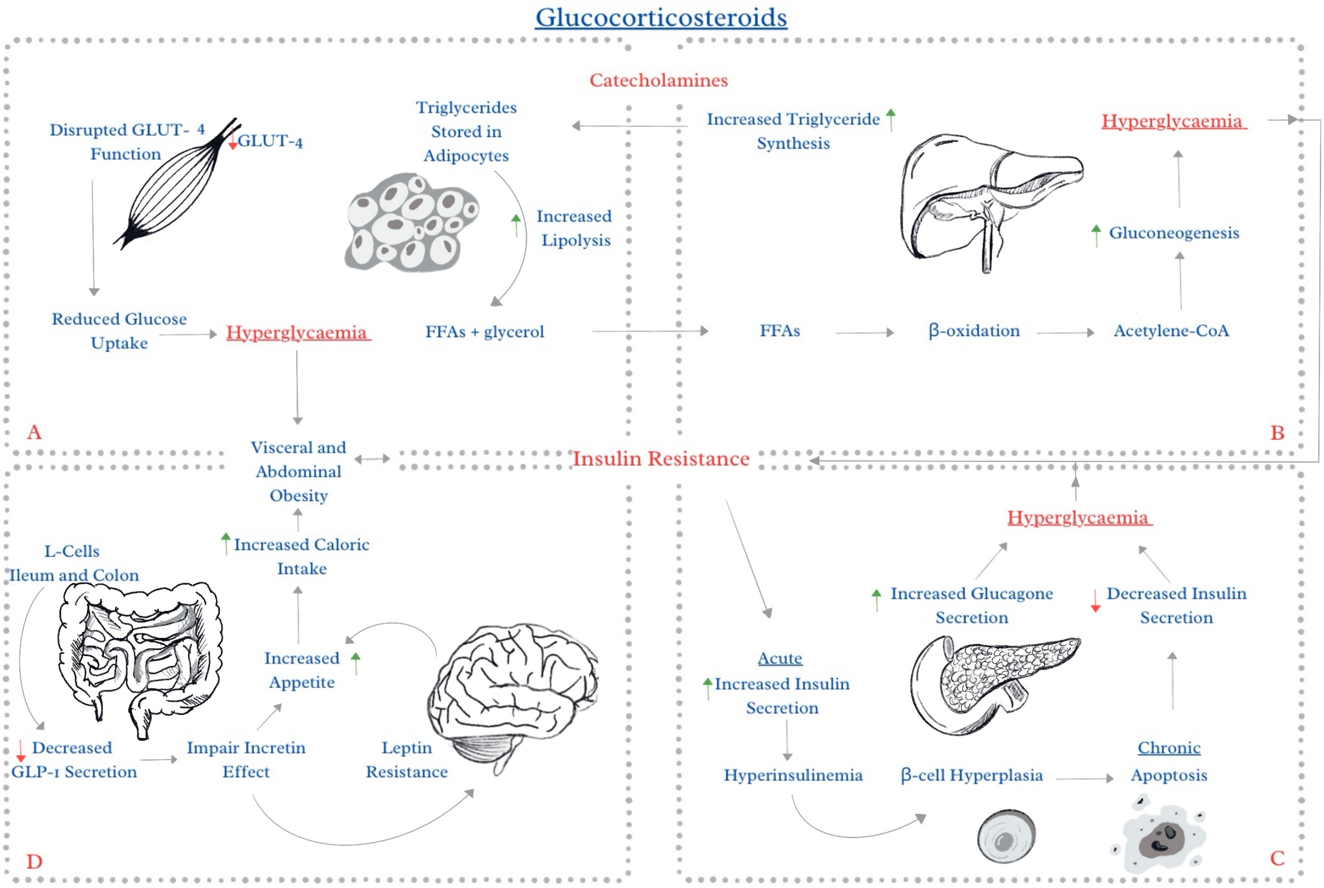

Figure S1: Metabolic Effects of Glucocorticoids.

Description: GCs increase the diabetogenic effect of catecholamines. A central feature of steroid-induced hyperglycaemia is insulin resistance in the liver, skeletal muscle, and adipose tissue, driven primarily by increased hepatic glucose production, lipolysis, and elevated circulating free fatty acids. **A:** GCs reduce peripheral glucose uptake by disrupting GLUT4 function in muscle and adipose tissue. They increase lipolysis, leading to an increase in FFAs. **B:** Excess FFAs entering the liver stimulate β-oxidation and increase acetyl-CoA levels, which activate gluconeogenesis. Due to increased insulin resistance, the suppression of gluconeogenesis by insulin is ineffective. **C:** At the pancreatic level, GCs directly impair β-cell function, promote endoplasmic reticulum stress, lipid accumulation, and apoptosis, leading to reduced insulin secretion. Simultaneously, GCs increase α-cell glucagon secretion, collectively exacerbating hyperglycaemia. **D:** The glucocorticoid receptor is present in GLP-1-producing cells, and its activation diminishes the secretory responsiveness of these cells. GCs impair incretin effect, increase appetite, leading to excessive caloric intake. GCs stimulate adipogenesis and fat redistribution, favouring visceral and abdominal adiposity. This vicious circle leads to significant hyperglycaemia and further increased insulin resistance. FFAs - free fatty acids; The figure was created by the author (A.J.).

2 SUPPLEMENTARY TABLE

Table S1: Summary of studies reporting efficacy and safety of GLP-1 RAs therapy in SIH and SID.

Study	Design	Population	GC Therapy	Intervention	Main Results
Suyama et al.[58] (2024, Japan)	Case series	8 patients with non-Hodgkin lymphoma; SIH	Prednisolone 100 mg ×5 days/cycle	Dulaglutide 0.75 mg/week	↓HbA1C decreased after GLP-1 RA initiation -median 5.9%;↓decrease in body weight (p=0.0006); ↓decreased glycoalbumin levels. (*source not peer-reviewed)
Pu et al. [38] (2022, China)	Cohort study	T2DM patients with malignant tumors (n=60 GLP-1 vs 60 insulin);SIH	N/A	Liraglutide 0.6–1.8 µg/d	↓Lower hospitalisation rate and shorter duration, Lower hypoglycemia rate ($p<0.05$) in liraglutide group; improved HbA1c, BMI and β cell function after 6 months of liraglutide treatment .
Zhang et al. [41] (2021, China)	Case report	SID; relapsing- remitting multiple sclerosis	Methylprednisolone 500 mg i.v. $\rightarrow$ 6 mg p.o.	Liraglutide 0.6 mg/d + insulin + metformin	Insulin discontinued after 2 months; HbA1c dropped substantially from 12.4% to 8.6%; decrease in body weight by 6.8 kg after 4 months; no significant gastrointestinal symptoms.
Uchinuma et al. [39] (2020, Japan)	Retrospective cohort	Hospitalised patients with SIH (n=38 GLP-1 vs n=38 insulin)	GCs including pulse therapy	Dulaglutide 0.75 mg/week + insulin	↓Decreased injection frequency ($p<0.001$) and total daily insulin dose ($p<0.01$) in the GLP-1 group; no increase in hypoglycemia or GI adverse events.
Hamasaki et al. [42] (2018, Japan)	Case report	SIH; Chronic hypersensitivity pneumonitis	Prednisolone 25mg/d p.o.$\rightarrow$ 15mg/d p.o.	Dulaglutide + mitiglinide + insulin	Discontinuation of insulin therapy; improved fasting and postprandial glucose, improvement in C-peptide and glucagon levels.

Study	Design	Population	GC Therapy	Intervention	Main Results
Matsuo et al. [59] (2013, Japan)	Case series	T2DM patients experiencing SIH; rheumatoid arthritis(2); myasthenia gravis(1); amyopathic dermatomyositis(1)	2:Prednisolone 7mg/d p.o. 1:Prednisolone 10mg/d 1:Prednisolone 2mg 2x/d	Exenatide 5 µg twice/d *one patient switched from liraglutide	Improved glycemic control, systolic blood pressure; decrease in low-density lipoprotein cholesterol and triglyceride levels; decrease in body weight in all cases.

Description: SIH - steroid-induced hyperglycaemia; GLP-1 RA - glucagon-like peptide-1 receptor agonist; T2DM - type 2 diabetes mellitus; N/A - not available; /d - per day; BMI - body mass index; SID - steroid-induced diabetes; i.v.-intravenous; p.o. - per os (oral administration);
