# Supplementary material for: Evaluation of GLP-1 receptor agonist therapy in the management of steroid-induced diabetes: a narrative review
Source: Front Clin Diabetes Healthc. 2026 Apr 10;7:1772391. doi: 10.3389/fcdhc.2026.1772391 (PMC13105962; doi:10.3389/fcdhc.2026.1772391)
Supplement: Supplementary file 2 [file DataSheet2.pdf]

Table S1: Summary of Studies Reporting Efficacy and Safety of GLP-1 RAs Therapy in SIH and SID.

Study	Design	Population	GC Therapy	Intervention	Main Results
Suyama et al.[58] (2024, Japan)	Case series	8 patients with non-Hodgkin lymphoma; SIH	Prednisolone 100 mg ×5 days/cycle	Dulaglutide 0.75 mg/week	↓HbA1C decreased after GLP-1 RA initiation -median 5.9%;↓decrease in body weight (p=0.0006); ↓decreased glycoalbumin levels. (*source not peer-reviewed)
Pu et al. [38] (2022, China)	Cohort study	T2DM patients with malignant tumors (n=60 GLP-1 vs 60 insulin);SIH	N/A	Liraglutide 0.6–1.8 μg/d	↓Lower hospitalisation rate and shorter duration, Lower hypoglycemia rate (p<0.05) in liraglutide group; improved HbA1c, BMI and β cell function after 6 months of liraglutide treatment .
Zhang et al. [41] (2021, China)	Case report	SID; relapsing- remitting multiple sclerosis	Methylprednisolone 500 mg i.v. → 6 mg p.o.	Liraglutide 0.6 mg/d + insulin + metformin	Insulin discontinued after 2 months; HbA1c dropped substantially from 12.4% to 8.6%; decrease in body weight by 6.8 kg after 4 months; no significant gastrointestinal symptoms.
Uchinuma et al. [39] (2020, Japan)	Retrospective cohort	Hospitalised patients with SIH (n=38 GLP-1 vs n=38 insulin)	GCs including pulse therapy	Dulaglutide 0.75 mg/week + insulin	↓Decreased injection frequency (p<0.001) and total daily insulin dose (p<0.01) in the GLP-1 group; no increase in hypoglycemia or GI adverse events.
Hamasaki et al. [42] (2018, Japan)	Case report	SIH; Chronic hypersensitivity pneumonitis	Prednisolone 25mg/d p.o.→ 15mg/d p.o.	Dulaglutide + mitiglinide + insulin	Discontinuation of insulin therapy; improved fasting and postprandial glucose, improvement in C-peptide and glucagon levels.

Study	Design	Population	GC Therapy	Intervention	Main Results
Matsuo et al. [59] (2013, Japan)	Case series	T2DM patients experiencing SIH; rheumatoid arthritis(2); myasthenia gravis(1); amyopathic dermatomyositis(1)	2:Prednisolone 7mg/d p.o. 1:Prednisolone 10mg/d 1:Prednisolone 2mg 2x/d	Exenatide 5 µg twice/d *one patient switched from liraglutide	Improved glycemic control, systolic blood pressure; decrease in low-density lipoprotein cholesterol and triglyceride levels; decrease in body weight in all cases.

Description: SIH - steroid-induced hyperglycaemia; GLP-1 RA - glucagon-like peptide-1 receptor agonist; T2DM - type 2 diabetes mellitus; N/A - not available; /d - per day; BMI - body mass index; SID - steroid-induced diabetes; i.v.-intravenous; p.o. - per os (oral administration);
